# A two-step method for fabricating large-area textile-embedded elastomers for tunable friction

**DOI:** 10.1098/rsos.181169

**Published:** 2018-10-31

**Authors:** Takuya Ohzono, Kay Teraoka

**Affiliations:** 1 Electronics and Photonics Research Institute, National Institute of Advanced Industrial Science and Technology (AIST), 1-1-1 Higashi, Tsukuba, Ibaraki 305-8565, Japan; 2 Human Informatics Research Institute, AIST, 1-1-1 Higashi, Tsukuba, Ibaraki 305-8566, Japan

**Keywords:** squeeze film, tunable friction, textile-embedded elastomer, large-area fabrication

## Abstract

Recently, shape-tunable wrinkles formed on an elastomeric sheet with a textile finely embedded in proximity to the surface have been developed for *in situ* control of friction depending on various situations. For their actual uses, sheets with a large area are desired. A key challenge on their fabrication is to overcome the non-uniformity of the vertical position of the textile embedded within the elastomeric sheet, which causes substantial reduction in the tunable range of friction. The defect originates from the increased difficulty, as the sheet area is scaled up, of squeezing a viscoelastic precursor liquid due to the use of a deformable elastomeric surface. Here, we report a new two-step method for a textile-embedded elastomeric sheet that avoids using the soft elastomeric surface on the squeezing process and requires post-joining to an elastomeric base sheet. The obtained sheet with a large area (180 × 180 mm), was uniform and showed a large change of friction on its strain-induced transformation between flat and wrinkled states. The relationship between the experimentally controllable parameters and the squeeze film hydrodynamics is theoretically discussed, which is generally applicable to precise embedding micro-objects at the elastomer surface.

## 1. Introduction

Soft materials such as rubber and plastics have been used in many tribological [1–3] applications with relatively non-severe contacts at the interfaces [4–16], including grips on tools and housewares. Some of the applications have a potential need to control the friction and/or adhesion properties depending on the usage situations; the *in situ* switchability of friction/adhesion of the materials surface has been desired. Recently, we have proposed the use of the shape-tunable surfaces to change the contact state at the interface [17–21], and thus, to tune the friction and/or adhesion simply by varying lateral strain applied to the surface. The shape tunability has been realized by the buckling-based surface wrinkling [22]. Wrinkles with sinusoidally wavy undulations spontaneously appear as a result of the strain-induced buckling of a hard surface layer formed on a soft elastic substrate. Using a certain type of shape-tunable wrinkles [23–25], various applications, other than tribological ones, including optical elements [26] and patterning of liquids [27,28], have been demonstrated. Especially, we have recently reported that a switchable hierarchical microstructure induced on a textile-embedded elastomer surface can remarkably change the friction depending on whether the surface is flat or wrinkled [19,20]; at low normal load, the friction coefficient on the flat surface of approximately 1.0 can be changed to approximately 0.1 by inducing the hierarchical microstructure.

The key surface feature to induce the large difference in friction coefficients between the flat and wrinkled states is the partially exposed fibres of the textile from the flat surface of the elastomer substrate [19,20]. Fabrication of the structure requires fine positioning of the textile sheet at the proximity surface of the elastomer substrate during the embedding process. The process includes problematic squeezing of the viscous liquid, the precursor of the elastomer to be cured.

For practical applications of the textile-embedded elastomer as versatile friction-tunable materials, a sheet with a larger area, e.g. at least 100 × 100 mm^2^ for grips grasped by a hand, would be desired. Toward this end, in the present study, we focus on the fabrication process of the sheet with a large area. In the following section, we start by describing the previous fabrication methods and show that a large-area sheet with a wide frictional tunability is difficult to obtain with these methods due to the topological inhomogeneity of the top surface. The inhomogeneity critically generates an adverse effect on the frictional property, reducing the tunable range of friction. As a solution, a new two-step method is presented, and we show that it enables the fine positional control of the embedded textile sheet at the proximity surface of the soft elastic substrate with a large area. The qualitative reasons why the two-step method works are discussed with a general theoretical model, which is related to the hydrodynamic process of squeezing a viscous liquid film [29–33].

## 2. Material and methods

### 2.1. One-step method

**Figure 1.**
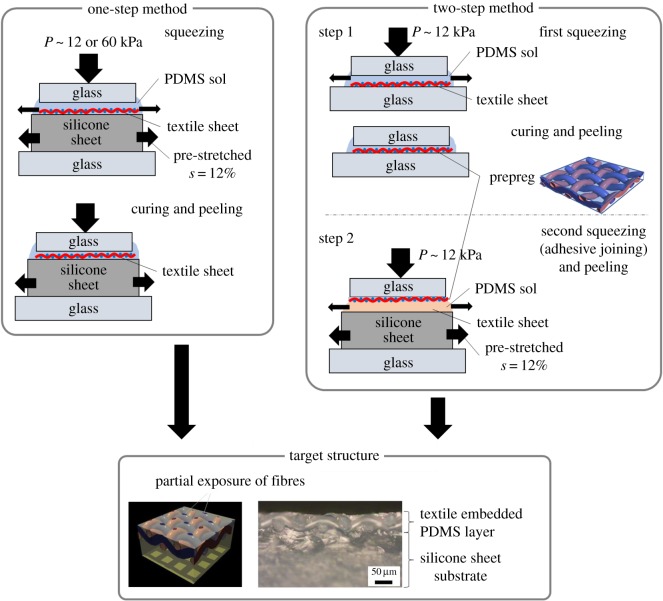
Fabrication methods of textile-embedded elastomers. The one-step (left) and two-step (right) methods are schematically shown.

Because the previous method [19] to fabricate textile-embedded elastomer surfaces with hierarchical structures includes one squeezing process (figure 1, left), it is called the ‘one-step method’ here. A plain-weave fabric (textile sheet) made of 35 µm diameter nylon-66 fibres with a distance between fibres of $d_{p}∼80\;\mu m$ (N-NO. 305, AzOne, Japan) was placed on a 2 mm thick uniaxially pre-stretched silicone rubber sheet (AzOne, Japan) at a strain *s* = 12%, and impregnated with a polydimethylsiloxane (PDMS) sol (the precured standard mixture of Sylgard 184, Toray-Dow Corning, Japan, the initial viscosity of approximately 3.9 Pa s). The samples were placed between supporting glasses and cured at room temperature (295 K) for 24 h under a constant pressure *P* ∼ 12 or 60 kPa applied normally to the surface, thus squeezing out the excessive PDMS sol. The samples were finally peeled from the supporting glasses. After peeling, the sample was subjected to internal compressive strain attributable to the release of the rubber sheet pre-stretching denoted by *s*. These conditions resulted in the buckling and wrinkling of the surface with a typical periodicity approximately 700 µm. In the previous studies, the sample area was less than the range of 20 × 20 mm. Here, it was enlarged to 180 × 180 mm.

### 2.2. Two-step method

The one-step method is divided into two squeezing steps (figure 1, right); the top layer of the textile-embedded elastomer sheet, which is called a prepreg sheet, is firstly prepared, and then, the prepreg sheet is adhered onto the pre-stretched elastomer sheet as follows. First, the textile sheet was placed on a 2 mm thick glass plate and impregnated with the PDMS sol. Another glass plate was placed on the textile sheet with the pressure *P* approximately 12 kPa to squeeze out the excessive sol of PDMS. The sample was cured at room temperature for 24 h under pressure. The prepreg was then peeled from the supporting glasses. After peeling, the prepreg applied with the PDMS sol on one side was placed on a 2 mm thick uniaxially pre-stretched silicone rubber sheet (AzOne, Japan) at strain *s* = 12%. The sample was cured at room temperature (295 K) for 24 h under a pressure *P* ∼ 12 kPa to join the prepreg and the silicone sheets together. After releasing the applied strain, the sample was subjected to internal compressive strain attributable to the release of the rubber sheet pre-stretching denoted by *s*. These conditions also resulted in the buckling and wrinkling of the surface with a typical periodicity approximately 700 µm, as is the case in the one-step method.

### 2.3. Characterizations

The surface topography was characterized using an optical microscope (BX-51, Olympus) and a laser confocal optical microscope (VK-9710, Keyence).

The sliding friction measurement was conducted using a pin-on-plate-type friction tester. A spherical glass indenter with a radius of approximately 12.7 mm was used. The normal load of approximately 1.5 N was adjusted by the mass placed above the indenter. Samples affixed to a rigid plate were moved in a direction at 10 mm s^−1^ for 1 s, and the force in the direction of motion was monitored with a force gauge (Tribogear, Type: 33, Heidon, Japan). Each measurement was repeated five times, and the averaged dynamic sliding friction force values divided by the normal load were analysed as the friction coefficients *μ*_d_. For wrinkled samples, the sliding was set in the direction perpendicular to the wrinkle grooves. To flatten the wrinkles, the uniaxial tensile strain of 12%, which was identical to *s*, was applied to the sheet. We focused on the difference between the *μ*_d_ values observed for wrinkled and flat states.

## 3. Results

### 3.1. One-step method (low-pressure squeezing)

**Figure 2.**
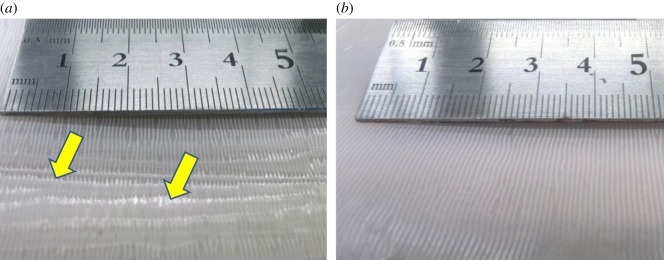
Photographs of surfaces of textile-embedded elastomers fabricated with the (*a*) one-step method with low applied normal pressure and (*b*) two-step method (see electronic supplementary material, figure S1 for whole sample). At the positions with the higher reflection, indicated by arrows on (*a*), there is no exposure of the fibre yarns to the surface; they are covered by the thin PDMS film due to the faulty squeezing process.

**Figure 3.**
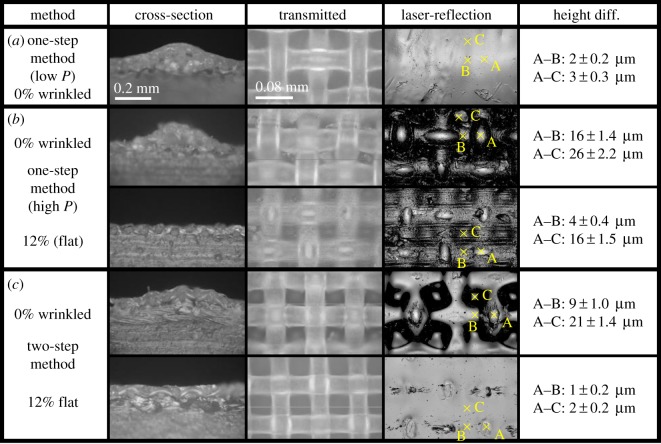
Optical microscopy images. The cross-sectional, transmitted and laser-reflection images are shown for samples fabricated with the (*a*) one-step method with low *P*, (*b*) one-step method with high *P* and (*c*) the two-step method. For (*b*) and (*c*), the flattened states under the external tensile strain of 12% are also shown. To evaluate the small undulations, the height differences between the points indicated by yellow crosses (A, B and C) are shown. Note that no exposure of fibre on (*a*) and non-negligible undulation on the flat state of (*b*) are observed.

**Figure 4.**
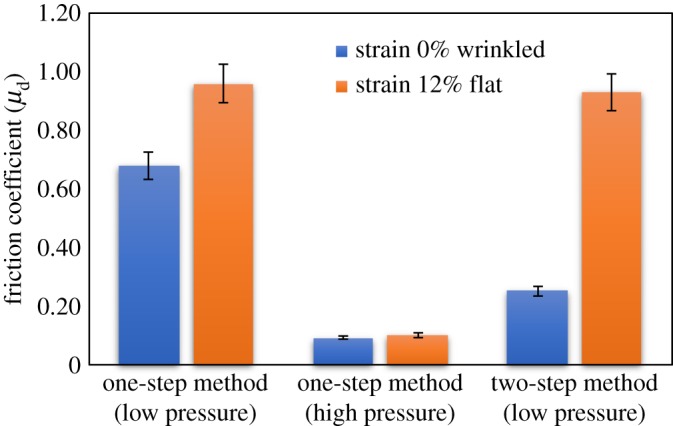
Frictional coefficients on the flat and wrinkled states of the textile-embedded elastomers fabricated with the one-step (at low and high applied normal pressures) and two-step methods.

Figure 2*a* shows a photograph of the sample surface, in which inhomogeneous light reflections are noted. The microscopy image taken at the higher reflection region is shown in figure 3*a*, indicating that the surface is covered by the cured PDMS; no exposure of the textile fibre to the surface is observed. As a result, both friction coefficients with both flat and wrinkled states show large values (figure 4) and the range of the variable friction becomes smaller than that observed previously [19]. The exposure of the textile fibres to the top surface is critically important to reduce the friction of the wrinkled state because they protrude and support the load with their small area under frictional sliding. When the textile fibres are covered with PDMS, the contact area can increase by deformation of the soft elastic PDMS resulting in the frictional increase. The results confirm that the fine positioning of the textile sheet at the proximity surface is primarily of importance for the present system and suggest that the one-step method with low *P* cannot provide such a sheet with the large area addressed here.

### 3.2. One-step method (high-pressure squeezing)

As the previous result suggests that the process of squeezing the PDMS sol was insufficient with low *P*, it was increased to *P* = 60 kPa to enhance squeezing. The resultant sample surface showed the small undesired undulation even when the sample was stretched with the strain of 12% to flatten the buckling-based wrinkles (figure 3*b*, bottom). As a result, the friction coefficients of both flat and wrinkled states showed small values, and thus, little difference between them indicating no frictional switchability (figure 4).

Here, we briefly discuss the mechanism for the formation of the undesired small undulations, which should be related to the larger normally applied pressure *P*. As the bulk modulus of nylon-66 is of the order of GPa, the textile sheet is pressed under *P* ∼ 60 kPa onto the soft elastic silicone surface (with the modulus of *E* ∼ 1 MPa) after squeezing out the excess PDMS sol. As the textile sheet originally has a characteristic topography due to the weave structure, the intersection points of the two fibres mainly support the normal pressure, and thus, may sink into the soft substrate concaving it locally. When the pressure is removed after curing PDMS, the silicone base tends to relax the concaved deformation causing the intersection points to push upward and protrude in the normal direction of the surface, relatively to the other position.

Assuming that the fibre indenting the silicone surface can be roughly approximated as a sphere with a diameter of *d*_f_ at the intersection point, the relative depression *D*_s_ can be estimated using the Hertzian contact theory [3]. The force between a hard sphere with a radius $R=\;d_{f}/2∼17.5\;\;\mu m$ and a soft and elastic half-plane with a modulus *E* ∼ 1 MPa yields

$$F=\;\frac{4}{3}ED_{s}^{3/2}R^{1/2}.$$

Here, *F* is estimated as the force applied on the single intersection point, which is located on each lattice unit of the textile with an area of $d_{p}^{2}∼80^{2}\;\mu m^{2}$. Then, *F* can be estimated as $Pd_{p}^{2}∼0.38\;\;\mathrm{mN}$, and *D*_s_ is estimated to be approximately 17 μm by using the relation

$$D_{s}={\left[\frac{3F}{4ER^{1/2}}\right]}^{2/3}={\left[\frac{3Pd_{p}^{2}}{4ER^{1/2}}\right]}^{2/3}.$$

This shows good consistency, considering the rough estimation, with the depth of the small undulation on the top surface having an observed value of $D_{s}∼16\pm 1.5\;\mu m$, which are found as the height difference at points A and C in figure 3*b*, bottom. As the relative depression should be minimized to ensure the large contact area, and thus high friction on the flattened state, the increase of applied pressure *P* on fabrication is inadvisable. Consequently, the results suggest that a new method other than the simple modification of the experimental parameters on the one-step method is required to fabricate the specified structure with a large area.

### 3.3. Two-step method

The optical images of the surfaces of the sheet fabricated by the two-step method are shown in figure 3*c*. The exposure of the textile fibre to the surface is confirmed (figure 3*c*). Moreover, the surface flatness has been achieved under the tensile strain of 12% in order to straighten the wrinkles (figure 3*c*, bottom). As shown in figure 4, the friction coefficients with flat and wrinkled states show large and small values, respectively, and the range of the variable friction coefficients becomes 0.2–0.9, which is comparable to the previously reported values [19] obtained for the smaller samples.

Although, as shown here, the present two-step method works well to obtain the textile-embedded elastomer sheet with a large area, the explanation and understanding of the background mechanism have not been discussed. In the next section, we clarify the key parameters that may cause the problems that resulted from the one-step method and the reasons for the success of the two-step method, with the aid of the squeeze film theory [29–33], which has long been developed for hydrodynamic lubrication problems.

## 4. Discussion

### 4.1. Squeeze film theory

Here, the squeeze film theory [29–33] is overviewed to qualitatively discuss the origin of the problems in the one-step method and the reason for the success of the two-step method. The squeeze film has long been known as one of the main problems of hydrodynamic lubrication, where the actions of viscous fluids to diminish friction and wear between solid surfaces are considered. In the present study, the squeezing process is required to impregnate the textile sheet with the PDMS sol and to press the textile sheet to the surface of the flat template plate. Although the lubrication is not the purpose of the present study, the squeeze film theory can be applied directly and the dominant parameters, which are important to tune the experimental conditions, can be clarified. The theory is based on the Navier–Stokes equations under the thin film approximation with low Reynolds' number *Re*, resulting in the Reynolds’ equation as follows.

### 4.2. One-step method (high-pressure squeezing)

**Figure 5.**
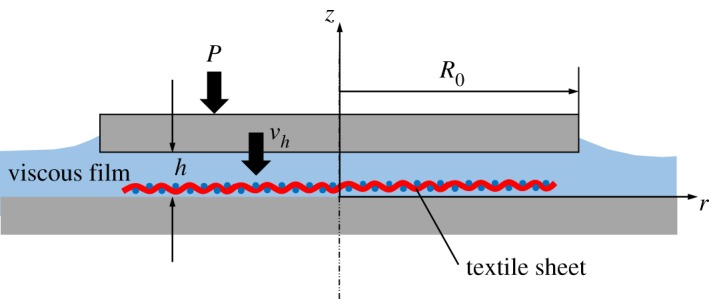
Schematic of the squeeze film system. Although the textile sheet is shown, the effect on the hydrodynamics is neglected in the theoretical treatment for simplicity.

The system considered here is schematically shown in figure 5, where the time-dependent film thickness is *h*, the characteristic length of the sample area is *R*_0_ and the local pressure is *p*, which is only dependent on the radial position *r*. Although the textile sheet is placed within the viscous film in the experiments, the effect on the fluid flow is neglected for simplicity. The initial thickness of the film between two substrates *h*_0_ at *t* = 0 decreases down to *h*_f_, which is the effective thickness of the textile, at *t* = *t*_f_, under the applied constant pressure *P*. To describe the system qualitatively, we assume here an axisymmetric circular system instead of the square one and the fluid density *ρ* and viscosity *μ* constants, which in a real system may change with time as cross-linking reactions proceed. The Navier–Stokes equations in cylindrical coordinates (*r*, *θ*, *z*) are written as follows:

4.1 $$\rho \left(\frac{\partial v_{r}}{\partial t}+v_{r}\frac{\partial v_{r}}{\partial r}+v_{z}\frac{\partial v_{r}}{\partial z}\right)=-\frac{\partial p}{\partial r}+\mu \left(\frac{\partial^{2}v_{r}}{\partial r^{2}}+\frac{1}{r}\frac{\partial v_{r}}{\partial r}+\frac{\partial^{2}v_{r}}{\partial z^{2}}-\frac{v_{r}}{r^{2}}\right)\;$$

and

4.2 $$\rho \left(\frac{\partial v_{z}}{\partial t}+v_{r}\frac{\partial v_{z}}{\partial r}+v_{z}\frac{\partial v_{z}}{\partial z}\right)=-\frac{\partial p}{\partial z}+\mu \left(\frac{\partial^{2}v_{z}}{\partial r^{2}}+\frac{1}{r}\frac{\partial v_{z}}{\partial r}+\frac{\partial^{2}v_{z}}{\partial z^{2}}\right)\;,$$

where *v_r_* and *v_z_* are the fluid velocity in the radial and the axial direction, respectively. According to the thin film condition *h* ≪ *R*_0_ and disregarding the inertia effects, the order magnitude of equation (4.2) is much smaller than that of equation (4.1) and some terms in equation (4.1) can be neglected, resulting in:

4.3 $$\frac{\partial p}{\partial r}=\;\mu \frac{\partial^{2}v_{r}}{\partial z^{2}}.$$

Integrating equation (4.3) twice with respect to *z* under the boundary conditions *v_r_* = 0 at *z* = 0 and *h* = 0, the radial flow velocity is derived as follows:

4.4 $$v_{r}=\frac{1}{2\mu }\frac{\partial p}{\partial r}(z^{2}-hz).$$

The related continuity equation for an incompressible fluid in cylindrical coordinates is as follows:

4.5 $$\frac{1}{r}\frac{\partial }{\partial r}(rv_{r})+\frac{\partial v_{z}}{\partial z}=0.$$

Substituting equation (4.4) into (4.5), and integrating that with respect to *z* from 0 to *h* under the boundary condition *v_z_* = 0 at *z* = 0 and $v_{z}=\;\frac{\partial h}{\partial t}=v_{h}$ at *z* = *h* leads to the Reynolds' equation in cylindrical coordinates as follows:

4.6 $$\frac{\partial }{\partial r}\left(rh^{3}\frac{\partial p}{\partial r}\right)=\;12\mu rv_{h}.$$

Here, we assume that squeeze surfaces are rigid, and the squeezing velocity is sufficiently small. The Reynolds’ equation is integrated using the boundary condition for the pressure gradient at the centre being zero; ∂p/∂r=0 at *r* = 0, resulting in

4.7 $$\frac{\partial p}{\partial r}=\frac{6\mu rv_{h}}{h^{3}}.$$

Neglecting the fluid inertia, the pressure at the periphery of the squeeze film is equal to the ambient pressure, zero; *p* = 0 at *r* = *R*_0_. Integration of equation (4.7) under this boundary condition yields

4.8 $$p=\frac{3\mu v_{h}}{h^{3}}(r^{2}-R_{0}^{2}).$$

The pressure difference at the centre and the periphery becomes $\Delta p=-(3\mu v_{h}/h^{3})R_{0}^{2}$. As we consider *v_h_* < 0, in which the film thickness *h* decreases with time, the positive pressure at the centre, which is proportional to the squeeze area, rises.

Integrating equation (4.8) over the squeeze surface gives the total load *W* as follows:

4.9 $$W=2\pi \int_{0}^{R_{0}}rp\;dr=-\frac{3\pi \mu v_{h}R_{0}^{4}}{2h^{3}}.$$

The experimentally controlled normally applied pressure *P* is expressed as follows:

4.10 $$P=\frac{W}{\pi R_{0}^{2}}=-\frac{3\mu v_{h}R_{0}^{2}}{2h^{3}}.$$

With this equation, equation (4.8) can be rewritten as follows:

4.11 $$p=2P\left[1-{\left(\frac{r}{R_{0}}\right)}^{2}\right],$$

which is time-independent under the constant pressure *P*.

Using boundary conditions for the initial and final film thickness: *h* = *h*_0_ at *t* = 0 and *h* = *h*_f_, which is the effective thickness of the textile, at *t* = *t*_f_, equation (4.10) is solved with respect to *h* yielding

4.12 $$h={\left(\frac{4P}{3\mu R_{0}^{2}}t+\frac{1}{h_{0}^{2}}\right)}^{-1/2}$$

and

4.13 $$t_{f}=\frac{3\mu R_{0}^{2}}{4P}\left(\frac{1}{h_{f}^{2}}-\frac{1}{h_{0}^{2}}\right).$$

Equation (4.13) indicates that the required duration to squeeze film is proportional to $R_{0}^{2}$; when the characteristic length scale *R*_0_ increases tenfold, *t*_f_ increases a hundred times. Practically, the cross-linking reaction in the present PDMS sol proceeds at room temperature, which means *μ* increases with time. Therefore, the increased *t*_f_ for the large sample area may easily cause the faulty squeeze and thus result in the inhomogeneous position of the textile sheet found in the result (figures 2*a* and 3*a*) obtained by the one-step method with low *P*.

Equation (4.13) also indicates that *t*_f_ decreases with increasing *P*. This supports that the one-step method with the higher *P* may result in the homogeneous position of the textile sheet. Indeed, the homogeneous squeezing has been attained with the higher *P* = 60 kPa. However, a higher *P* can cause another problem of dents of the soft surface due to the stress focusing on the intersection points of the textile as described before. As another way to reduce *t*_f_, the use of a PDMS sol with the lower viscosity *μ* is considered. However, it is difficult to decrease it without changing other mechanical parameters of the cured PDMS and requires fine-tuning of the polymeric components in the PDMS sol. Consequently, we theoretically find that the one-step method is not appropriate for obtaining the specified structure across large areas.

## 5. Conclusion

We report a new two-step method to fabricate the shape-tunable textile-embedded elastomeric sheet with an area larger than 100 × 100 mm^2^ and explain our observations with a mechanism that governs the dynamics of the squeeze film problem. With this method, the textile sheet can be embedded in proximity to the surface of the elastomeric substrate with a high positional precision over a large area. Discussion using the theoretical model for the squeeze film unveils the dynamics of the squeezing process and the adverse effects of using the soft elastomeric surface as the squeezing plate. The proposed method has applications to processes of precise embedding general porous sheets including textiles and micro-objects (filler particles) in proximity to the surface of the elastomeric substrate, illustrating how relevant parameters, such as squeezing pressure, area, viscosity of squeezed fluid and moduli of substrates, and embedded materials, can be exploited to tailor the final embedded states.

## Supplementary Material

Photograph of surface of textile-embedded elastomer fabricated with two-step method

## Data accessibility

All data used in this research are included in the figures in the main article and in the electronic supplementary material.
