# Supplementary material for: A two-step method for fabricating large-area textile-embedded elastomers for tunable friction
Source: R Soc Open Sci. 2018 Oct 31;5(10):181169. doi: 10.1098/rsos.181169 (PMC6227999; doi:10.1098/rsos.181169)
Supplement: Photograph of surface of textile-embedded elastomer fabricated with two-step method [file rsos181169supp1.docx]

**Supplementary Information *for***

A two-step method for fabricating large-area textile-embedded elastomers for tunable friction

**by**

**Takuya Ohzono and Kay Teraoka**

Supplementary Fig. S1


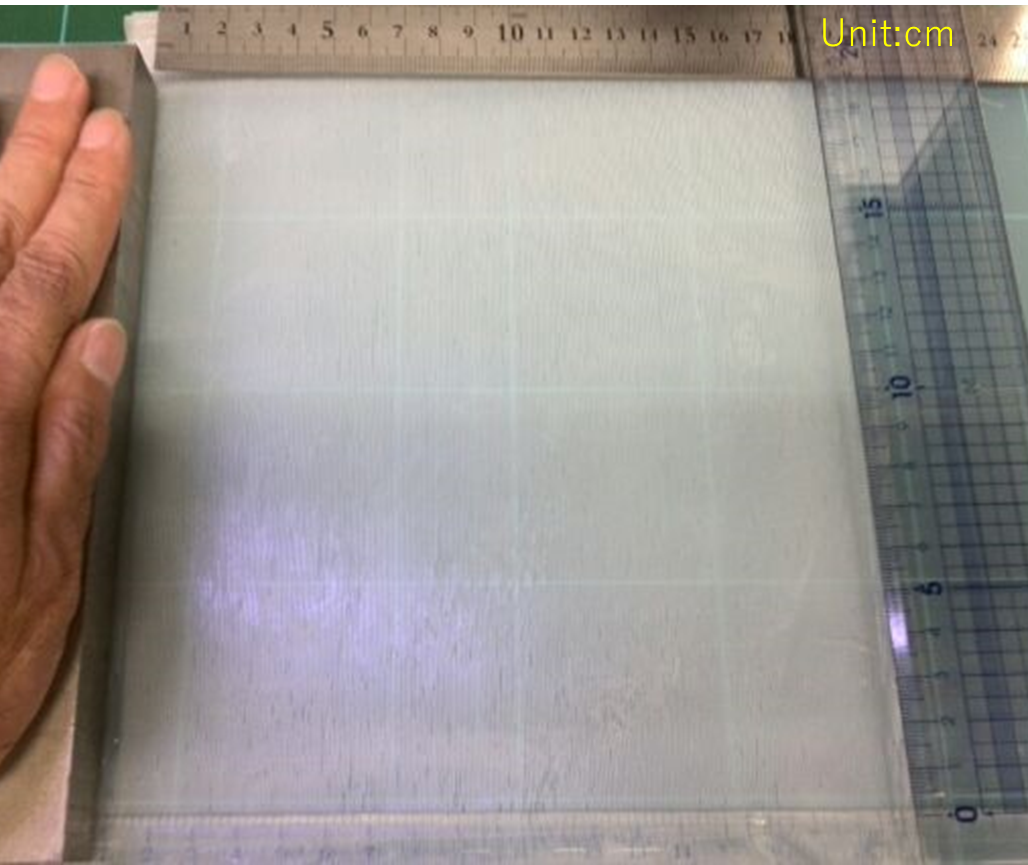


**Supplementary Figure S1. Photograph of surface of textile-embedded elastomer fabricated with two-step method.** The obtained surface area was 180 mm × 180 mm.
